# PHTLS ® (Prehospital Trauma Life Support) provider courses in Germany – who takes part and what do participants think about prehospital trauma care training?

**DOI:** 10.1186/1752-2897-8-7

**Published:** 2014-07-07

**Authors:** Christian B Frank, Christoph G Wölfl, Aidan Hogan, Arnold J Suda, Thorsten Gühring, Bernhard Gliwitzky, Matthias Münzberg

**Affiliations:** 1 Department of Orthopedic and Trauma Surgery – Clinic Mittelbaden, Dr. Rumpf Weg 7D 76530 Baden Baden, Germany; 2 Department of Trauma and Orthopedic Surgery – BG Trauma Center Ludwigshafen, Ludwig-Guttmann-Straße 13, D 67071 Ludwigshafen am Rhein, Germany; 3 German Association of Emergency Medical Technician (DBRD), Im Schlangengarten 52, D 76877 Offenbach/Queich, Germany; 4 PHTLS Research Group Europe (PERG), Im Schlangengarten 52, D 76877 Offenbach/Queich, Germany

**Keywords:** PHTLS, Interprofessional team training, Professional formation, Trauma care

## Abstract

**Background:**

The goal of this study was to examine PHTLS Provider courses in Germany and to proof the assumption that formation of physicians and paramedics in prehospital trauma care can be optimized.

**Methods:**

PHTLS participants were asked to fill out standardized questionnaires during their course preparation and directly after the course. There were some open questions regarding their professional background and closed questions concerning PHTLS itself. Further questions were to be answered on an analog scale in order to quantify subjective impressions of confidence, knowledge and also to describe individual levels of education and training.

**Results:**

247 questionnaires could be analyzed. Physicians noted significant (p < 0.001) more deficits in their professional training than paramedics. 80% of the paramedics affirmed to have had adequate training with respect to prehospital trauma care, all physicians claimed not to have had sufficient training for prehospital trauma care situations at Medical School. Physicians were statistically most significant dissatisfied then paramedics (p < 0.001). While most participants gave positive feedback, anesthetists were less convinced of PHTLS (p = 0.005), didn’t benefit as much as the rest (p = 0.004) and stated more often, that the course was of less value for their daily work (p = 0.03). After the course confidence increased remarkably and reached higher rates than before the course (p < 0.001). After PHTLS both groups showed similar ratings concerning the course concept indicating that PHTLS could equalize some training deficits and help to gain confidence and assurance in prehospital trauma situations. 90% of the paramedics and 100% of the physicians would recommend PHTLS. Physicians and especially anesthetists revised their opinions with regard to providing PHTLS at Medical School after having taken part in a PHTLS course.

**Conclusion:**

The evaluation of PHTLS courses in Germany indicates the necessity for special prehospital trauma care training. Paramedics and physicians criticize deficits in their professional training, which can be compensated by PHTLS. With respect to relevant items like confidence and knowledge PHTLS leads to a statistically significant increase in ratings on a visual analogue scale. PHTLS should be integrated into the curriculum at Medical School.

## Background

In a recent paper Johansson et al. showed that there might be a decrease in mortality rates after PHTLS training of ambulance caregivers in Sweden [1]. In addition there are data that there is a benefit for trauma patients not only in low- and middle-income countries after inauguration of standardized treatment strategies like PHTLS [1-18]. But there is still no evidence that mortality rates really can be decreased after PHTLS training [10,19,20]. However simulated trauma care team training and especially PHTLS is still going strong and succeeded in creating confidence and knowledge in participants [1,9-15,20-25].

Regarding PHTLS, in high-income countries there are still some skeptics and the course concept is sometimes under discussion, most especially in older trauma caretakers. Probably because it is so difficult to determine parameters that show in a fundamental way that we can save lives with PHTLS [1-6,10,19]. Nevertheless, dealing with PHTLS a lot of positive aspects have been shown and instructors like PHTLS providers are convinced that it not only “works” [25,26].

After PHTLS trauma team trainings negative attitude normally changes into positive and word-of-mouth recommendation seems to be the most meaningful reason for the overwhelming success [24].

In Europe and especially Germany prehospital trauma care belongs together to paramedics, physicians, firefighters, first responders and others. That is why PHTLS is offered to all professional players in the field. These different professional groups probably have different trauma loads and we assume that e.g. paramedics have more often to deal with severely injured patients and maybe have a different opinion towards PHTLS compared to physicians [7,16,27,28].

As far as we know there is yet no paper that describes the situation for Germany and deals with questions like the professional background of course participants and what they do think about their personal performance and training so far, before and after a PHTLS course. We also do not know whether experienced learners or rookies join the program. Thus the goal of this study was to examine the diversity of PHTLS courses in Germany and to proof the assumption that even from a professional’s viewpoint formation in prehospital trauma care can be optimized.

## Material and methods

After institutional approval (No. 837-032-11 (7574)) we asked all PHTLS participants in Germany over a period of 6 months (august 2011 – december 2011) to fill out standardized anonymously questionnaires during their course preparation and directly after the course.

During the investigated period 247 evaluation sheets of in-house and open courses were analyzed.

There were some open questions regarding their professional background and also closed questions concerning PHTLS itself. Further questions were to be answered on an analog scale ranging from 0 to 10 in order to quantify subjective impressions of confidence, knowledge and also to describe individual education and training.

Incomplete data sets were excluded from the analysis. For demographic data analysis all evaluation sheets were taken together and percentages were calculated. To show the discrepancy in opinions and their development during the PHTLS courses pre- and post-course evaluations were compared.

### Statistics

Statistical calculations were performed with the help of the Institute for Medical Biology and Informatics of the University of Heidelberg. The Chi Square Test and the Mann-Whithney-U-Test was used to detect any statistical significance concerning the results of the pre- and post-course evaluation and also statistical differences in subgroups of physicians and paramedics. A p-value < 0.05 was considered to be statistically significant.

## Results

### Demographic data

The participants could be divided into three major groups: physicians, paramedics and firefighters (Table 1). As all 17 firefighters had additional training (15 were paramedics, 2 were physicians, again one of these with paramedic training) only two groups consisting of physicians and paramedics (ratio nearly 3:4) were formed for further investigation.

**Table 1:** PHTLS course participants

	**♀**		**♂**		**Not applicable**	**∑**	
	**n**	**%**	**n**	**%**	**n**	**n**	**%**
**Paramedics/emergency medical services**	7	6	106	94	12	125	51
Paramedics with additional intensive care training/hospital nurse			2				
**Physicians**	29	29	70	71	6	105	42
Physicians with additional paramedic training	1		12			1	
**Fireworkers**			17	100		17	7
fireworkers with additional paramedic training			15				
Physicians employed at the fire department (1 with additional paramedic training)			2				
**∑**	36	16	193	84	18	247	100

229/247 evaluated sheets contained information concerning gender. Only 36 participants were female (16%). The majority of these were female physicians. 59% were anesthetists. There were no female firefighters.

### Professional situation

**Figure 1:**
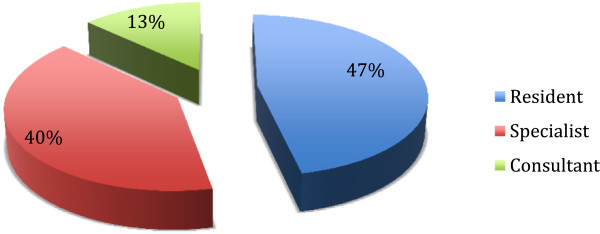
Physician - working status.

**Figure 2:**
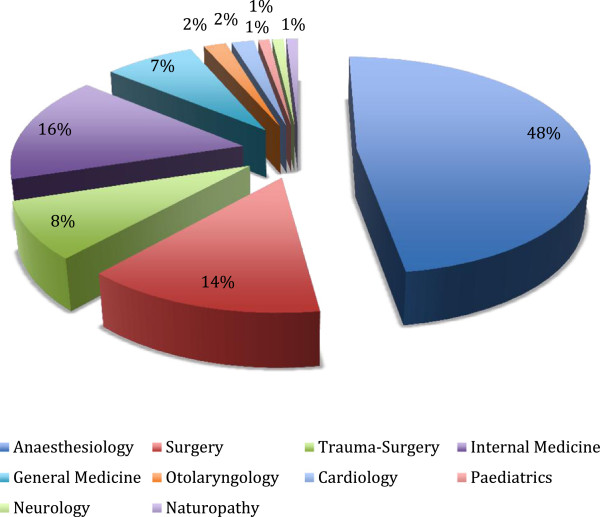
Physicians - specialties.

More than half of the physicians were specialists or specialist-consultants, 47% were residents. (Figure 1) 51% of the participating physicians were anesthetists, 9% were trauma surgeons. Apart from the over-representation of anesthetists the distribution of specialties resembled the normal range of specialties (Figure 2).

**Figure 3:**
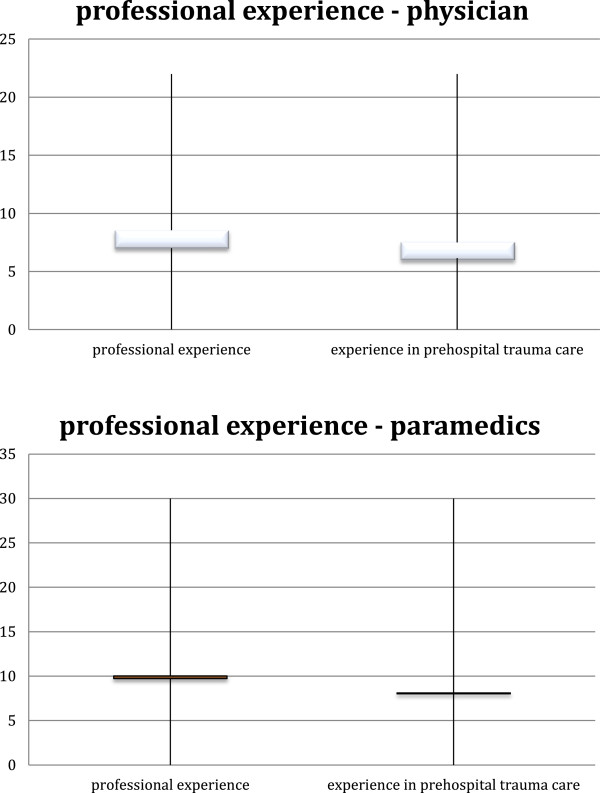
Professional experience overall and in prehospital trauma care (in years).

Table 2 provides an overview of the different day-to-day working life of all the participants. Most of them were involved in different areas of trauma care like emergency rooms, intensive care transportation and rescue helicopter teams. Some were instructors, teachers or worked in administration of emergency medical services like dispatch centers. A remarkable number of more than 60 participants were involved in mass-casualty scenarios (chief executive physicians, paramedics and members of the incident command) or were responsible for personnel.Although Physicians and paramedics had different professional experience regarding working years no statistical significance could be found in general (p = 0.483) and more specifically in pre-hospital trauma care (p = 0.088) (Figure 3).

**Table 2:** Every day work life of the course participants

**Function**	**Number**
Paramedics/emergency medical services	140
Emergency physician	107
Intensive care transportation^1^	87
Teacher/trainer/tutor	68
Helicopter crew member^1^	66
Course instructor	48
Chief executive physician	28
Chief executive paramedic	27
Incident command	24
Dispatch center/administration	22
Others	11

^1^either paramedic or physician.

### Professional training with regard to prehospital trauma care

Physicians noted significant (p < 0.001) more deficits in their professional training than paramedics: While 80% of the paramedics affirmed to have had adequate training with respect to prehospital trauma care, all physicians claimed not to have had sufficient training for prehospital trauma care situations at medical school. On the visual analog scale (range from 0 to 10) the ratings concerning the professional trainings were once more different. Again physicians were statistically most significant dissatisfied than paramedics (p < 0.001). Although physicians did not have high expectations towards their training in prehospital trauma care, their expectations still could not be met (p < 0.001). Paramedics gave more positive answers concerning their professional training especially in terms of practical skills and personal benefit through training (p < 0.001). Physicians gave the lowest scale ratings concerning their knowledge, self-confidence and their ability of living up to the requirements of treating trauma patient adequately under prehospital conditions immediately after having graduated from Medical School respectively in their first postgraduate years (p < 0.001). With increasing work experience both groups showed more confidence and assurance prior to the PHTLS courses becoming apparent in their positive scale ratings (7.4 versus 7.18) with no significant difference (p = 0.338).

Most paramedics would propose providing PHTLS courses for medical students at medical school as opposed to only 86% of the physicians. With regard to the physicians anesthetists were most skeptical against PHTLS. Nevertheless some other subgroups gave resembling answers concerning distinct items. For instance 25% of the trauma surgeons would not favor a course like PHTLS at Medical School. But anesthetists were less convinced of PHTLS (p = 0.005), didn’t benefit as much as the rest (p = 0.004) and stated more often, that the course was of less value for their daily work (p = 0.03).

Physicians more often than paramedics propose voluntary participation in PHTLS courses for professionals. Again the group of anesthetists more often opposes obligatory participation in PHTLS courses. Nevertheless both groups would recommend obligatory participation in PHTLS courses for pre-hospital trauma care providers.

### Evaluation after PHTLS courses

An interesting result, which is illustrated on the visual analogue scale, was that participants’ self-assessment with respect to confidence and assurance in the prehospital trauma situations was lower in retrospect than it had been prior to their course participation (p < 0.001). After the course confidence etc. increased remarkably and reached higher rates than before the course (p < 0.001). After PHTLS both groups showed similar ratings concerning the course concept indicating that PHTLS could equalize some training deficits and help to gain confidence and assurance in prehospital trauma situations. Participants were convinced by PHTLS and 90% of the paramedics compared to 100% of the physicians would recommend PHTLS to colleagues. Still anesthetists were skeptical in regard to the importance of PHTLS for their personal situation. 3 out of 5 physicians thought that the continuing education offers in prehospital trauma care were sufficient. 9 out of 60 paramedics concurred with this opinion. But interestingly physicians and especially anesthetists revised their opinions with regard to providing PHTLS at Medical School after having taken part in a PHTLS course. Only 6% of the physicians and 2% of the paramedics objected to the necessity for PHTLS training at Medical School.

## Discussion

The death rate of people in their most productive years is higher due to trauma than to cancer. Considering that the vast socio-economic consequences in modern ageing societies attention has turned to optimizing trauma care [7,8,11,12,14,15,21,23]. The aim is to save life and productive years. Unfortunately no strong data exist demonstrating the positive effects of PHTLS in high-income countries with regard to mortality and morbidity after trauma [1,10,19]. Nevertheless despite the absence of scientific proof, PHTLS courses are provided worldwide enjoying increasing popularity. We wanted to know what course participants in Germany think concerning their professional training in prehospital trauma care and why courses such as PHTLS are so popular. Furthermore we wanted to examine if PHTLS affected confidence and self-estimation as described in previous publications for other training concepts [2-5,9,11-13,15,18,22-24].

### Demographic data

It still remains unclear whether the participants of evaluated PHTLS courses represent the average of prehospital trauma care providers in proportion and knowledge in Germany and probably this will be unclear in the future [9]. In order to achieve and wide diversity in participants in-house and open courses were mixed fort his study. This fact may explain the relatively large contingent of emergency physicians. Another reason may also be that in Germany emergency physicians normally only participate in emergency service a few days per month compared to paramedics who participate in trauma care on a daily basis. This increases the total number of physicians required to provide this service. Less than 3% participated involuntarily in the courses. This implies a higher than average motivation and may determine a bias towards the pleasing results for PHTLS enthusiasts [24].

Our data suggests that prehospital trauma care in Germany is for the most part provided by male paramedics, firefighters and physicians. Females are more rare and are mainly anesthetists (Table 1). While most participating physicians are anesthetists those who treat trauma patients operatively under hospital conditions (surgeons) were rarely found at these courses.

In Germany numerous male physicians have additional paramedic training likely owing to working as civil servants as an alternative to military service. It is known that many of these young men continued to work in emergency medicine. Many decided to become physicians or even emergency doctors. As emergency medicine is an integral part of the training to become anesthetist it is not surprising that you find more anesthetists than for instance surgeons in this field of expertise. Regional differences are due to local structures and depend on the possibility of partaking in emergency medicine either alongside your own regular career or as a full time profession.

In contrast to other papers our results demonstrate less diversity and an extremely high level of participants’ expertise [9]. But this does not necessarily represent the average of expertise in Germany and may also have influenced the results of the questionnaires. It is likely due to the fact that when PHTLS was started in Germany participants were mainly experienced rather than beginners. This especially applies to paramedics. A further reason may be the structure of in-house courses. These courses were provided for rescue helicopter teams and emergency medicine centers with a higher number of well-trained specialists with many years of experience in trauma care.

### Professional training with regard to prehospital trauma care

It is more or less a critical feature that physicians state that prehospital trauma care training is not adequately represented at Medical School. Only 20% of paramedics agree with this opinion for their professional education. However, more paramedics than physicians propose PHTLS to be offered at Medical School. This may be an indirect proof of the physicians’ opinion not to be trained adequately.

PHTLS attempts to teach evidence based medicine in a field that has not yet gained particular scientific interest compared to e.g. heart failure or resuscitation and maybe is only in the focus of a small group of trauma interested care providers [1,8,10,12,14,15,23,27-29]. This may explain why some specialists like anesthetists are skeptical towards the teaching concept. Our data cannot show that anesthetists are skeptical per se however other specialists have the same opinion. But anesthetists’ opinions differed statistically significant more often from all the others maybe indicating that they have a more differentiated point of view on their own professional career. Again anesthetists were the largest group of physicians that makes the amount of less enthusiastic answers understandable. Nevertheless it seems obvious that this group has yet to be convinced.

In discussions during the courses physicians argued that not every student wants to work in trauma care and therefore special trainings at Medical School should be offered only voluntarily. Moreover, emergency physicians in Germany, who work in prehospital trauma care have to complete a training consisting of a placement in an intensive care unit or an emergency department, a special prehospital trauma care course and up to 50 emergency treatments of life threatening situations under the supervision of an experienced emergency physician. The same argument explains why physicians should have the opportunity to take part at these courses voluntarily. In contrast, after the course, almost all physicians stated that PHTLS should be taught at Medical Schools and would recommend PHTLS to their colleagues. This indicates very clearly that PHTLS can fulfill the expectations of its participants towards professional training and medical teaching.

Why are paramedics interested in PHTLS being taught at Medical School? It is not only the team approach that is trained. The level of uncertainty and lack of confidence is highest in physicians right after medical school and in the first years of their professional training. This subgroup may benefit most from PHTLS. If the principles of trauma care were trained as early as at Medical School along with PHTLS, theoretical and practical skills may improve and team formation could become a regular aspect in prehospital trauma care [8,12,13,20,21,23]. Nevertheless the importance of the team leader cannot be denied [9,12,13,20,22,24]. In addition to teamwork the ABCDE algorithm helps doctors to deal with unknown trauma situations and their responsibility as team leaders [9,12,13].

As afore-mentioned participants’ self-assessment with respect to confidence and assurance in the prehospital trauma situations was lower in retrospect than it had been prior to their course participation. This disparity may be owed to participants’ improved practical and theoretical skills through PHTLS training. On the other hand this bias is known from other authors and the optimistic self-estimation must not go along with improved knowledge and skills [30-32].

All participants, considering their level of pre-existing expertise, admitted to benefiting from PHTLS courses. In addition, after the course both professional groups rated similarly with respect to individually benefit, teaching manner and PHTLS’s impact on daily work.

One may counter, that these enthusiastic statements are due to a pleasant and interesting team building weekend and therefore have a bias shifting the results to a much better outcome. Again the participating quota of the survey could not be clarified because the anonymized questionnaires were collected at 2 time points and participation was voluntary. Nonetheless, the increase in confidence, certainty and safety was statistically significant (p < 0.001). Regarding the possible bias, the results may demonstrate at least how adult education should be conducted [24].

Another obvious limitation to the study is that the results cannot be objectified reliably like ongoing increase in knowledge, lasting reduction of prehospital rescue time and significant decrease of mortality is not proved [27,28].

It still is a hope that the reduction of prehospital time after trauma, which is documented in the German “TraumaRegisterDGU®” for 2011 is due to the increasing percentage of PHTLS graduates. (http://www.traumaregister.de) Unfortunately this parameter is not merely a result of team training and a standardized approach to trauma patients but of various factors.

## Conclusion

The evaluation of PHTLS courses in Germany indicates like in other countries the necessity for special prehospital trauma care training in Germany [1,9,10,12,13,20,24]. Both paramedics and physicians criticize deficits in their professional training that can be compensated by PHTLS. With respect to relevant items like confidence and knowledge PHTLS leads to a statistically significant increase. Furthermore, after a PHTLS course all paramedics and physicians rated on an equal level indicating homogeneity. From a professional’s point of view, PHTLS should be integrated into the curriculum at Medical School. In order to prove the positive effect of PHTLS from educational perspective further investigations should focus on the sustainability of PHTLS courses [1,12,27,28]. To prove that PHTLS has a positive effect on mortality rates in high-income countries remains difficult due to the vast amount of determining factors and patients that have to be included in probably a nationwide study [8,12,13,33].

## Competing interests

CF, CW and MM are medical course directors PHTLS Germany and receiving an expense allowance for PHTLS courses. CW is further the national director of PHTLS. BG is the chair of PHTLS. BG is further PHTLS instructor in Germany and receiving an expense allowance for PHTLS courses.

AH, AS and TG declare to have no competing interests.

## Authors’ contributions

CF, MM, CW designed, conduct the study and acquisition the data. CF, CW, AS, TG analyzed the data. CF, MM, AH prepared the manuscript. All authors have given the final approval of the paper.
